# Oxidative Stress-Mediated Skeletal Muscle Degeneration: Molecules, Mechanisms, and Therapies

**DOI:** 10.1155/2016/6842568

**Published:** 2015-12-22

**Authors:** Min Hee Choi, Jin Rong Ow, Nai-Di Yang, Reshma Taneja

**Affiliations:** ^1^Department of Physiology, Yong Loo Lin School of Medicine, National University of Singapore, Singapore 117597; ^2^NUS Graduate School for Integrative Sciences and Engineering, National University of Singapore, Singapore 117597

## Abstract

Oxidative stress is a loss of balance between the production of reactive oxygen species during cellular metabolism and the mechanisms that clear these species to maintain cellular redox homeostasis. Increased oxidative stress has been associated with muscular dystrophy, and many studies have proposed mechanisms that bridge these two pathological conditions at the molecular level. In this review, the evidence indicating a causal role of oxidative stress in the pathogenesis of various muscular dystrophies is revisited. In particular, the mediation of cellular redox status in dystrophic muscle by NF-*κ*B pathway, autophagy, telomere shortening, and epigenetic regulation are discussed. Lastly, the current stance of targeting these pathways using antioxidant therapies in preclinical and clinical trials is examined.

## 1. Sources of Reactive Oxygen Species

Reactive oxygen species (ROS) are highly reactive oxygen-containing molecules that are natural by-products of eukaryotic cellular metabolism [1, 2]. Primary sources of ROS in cells are the membrane-bound NADPH oxidase complex and the electron transport chain (ETC) in the mitochondria [1–4]. NADPH oxidase is a protein complex that is comprised of a membrane-bound NOX protein, p22^phox^, p47^phox^, p67^phox^, p40^phox^, and the small GTPases Rac1 or Rac2. There are five homologues of the NOX protein, each of which shows distinct tissue-specific expression patterns. For instance, NOX1 is expressed in the colon, smooth muscle, and placenta, while NOX2 is expressed in phagocytes, skeletal muscle, and neurons [3]. Upon activation, NADPH oxidase utilizes NADPH as an electron donor to produce the ROS superoxide (^∙^O_2_^−^) [3]. In contrast, production of ROS at the mitochondrial ETC is an unintended consequence of inefficiencies in the transfer of electrons between the complexes. The major sites of ROS leakage are believed to be complexes I and III, although other components of the ETC also have considerable contribution to the overall amount of ROS produced at the mitochondria [4]. Given that excessively high amounts of intracellular ROS have dire repercussions, it is unsurprising that each step of ROS generation is tightly detoxified by a line of antioxidant enzymes. For example, ^∙^O_2_^−^ is converted to hydrogen peroxide (H_2_O_2_) by the antioxidant enzyme superoxide dismutase (SOD). H_2_O_2_ is then reduced to water either by glutathione peroxidase or catalase. Glutathione peroxidase is a cytoplasmic selenoprotein that reduces H_2_O_2_ as well as other hydroperoxides to water by oxidizing reduced glutathione (GSH) to oxidized form (GSSG) [5]. Catalase is a ubiquitously expressed protein mainly localized in peroxisomes and it is able to reduce H_2_O_2_ efficiently [6]. Apart from the ETC, monoamine oxidases (MAO) in mitochondria also participate in substantial ROS production. MAO catalyzes oxidative deamination of neurotransmitters, dietary amines, and H_2_O_2_ [7]. Reactive nitrogen species (RNS) also contribute to cellular oxidative stress. Nitric oxide synthase (NOS) converts L-arginine to L-citrulline and generates nitric oxide (^∙^NO). ^∙^NO is required for various signaling pathways such as stimulation of guanylate cyclase to increase cyclic GMP (cGMP) production. Nevertheless, ^∙^NO is able to react with ^∙^O_2_^−^ to produce highly reactive peroxynitrite (ONOO^−^) and induce oxidative stress injury [8].

Emerging evidence suggests that ROS mediate several intracellular signaling pathways required for physiological functions [9]. However, excess ROS production, either due to overproduction of ROS or failure to remove them by cellular antioxidant defenses, is one of the detrimental attributes in various human pathologies such as ischemic disorders, cancer, degenerative diseases, and cellular aging [2, 7–12].

## 2. Topography of ROS Production in Skeletal Muscle

**Figure 1:**
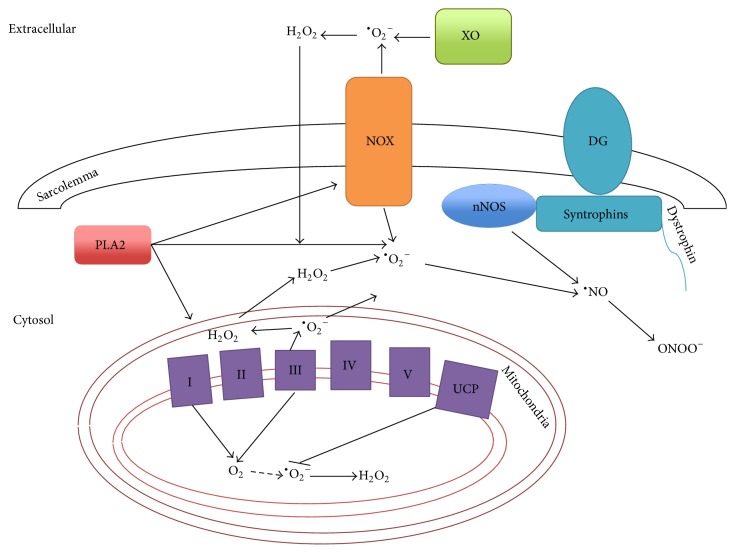
Multiple sites of ROS and RNS production are present in skeletal muscle cells. The network of different proteins that leads to ROS/RNS production in the extracellular, cytosolic, and mitochondrial compartments of skeletal muscle cell is illustrated. XO: xanthine oxidase; NOX: NADPH oxidase; DG: dystrophin-glycoprotein; nNOS: neuronal nitric oxide synthase; PLA2: phospholipase A2; I–V: complexes of electron transport/oxidative phosphorylation; UCP: uncoupling protein; O_2_: oxygen; ^∙^O_2_^−^: superoxide; H_2_O_2_: hydrogen peroxide; ^∙^NO: nitric oxide; ONOO^−^: peroxynitrite.

ROS have long been associated with both physiology and pathology of skeletal muscle. Production of ROS promotes muscle adaptation to exercise [13]. ROS are generated at multiple subcellular locations in skeletal muscle (Figure 1). Like other nonmuscle tissues, mitochondrial ETC complexes I and III are considered to be the major sites of ROS production in skeletal muscle [4]. Upon contraction, a 100-fold increase in total mitochondrial oxygen consumption only leads to 2- to 4-fold increase in total ROS production [14]. This is partly attributed to attenuation of mitochondrial ROS production by the uncoupling proteins (UCPs) [15], but it also implicates the presence of other ROS production sites apart from mitochondria. Potential nonmitochondrial sources of ROS production in skeletal muscle contraction include NOX, Xanthine oxidase (XO), Phospholipase A2 (PLA2), and NOS. NOX2, the main NOX isoform expressed in skeletal muscle, is located within the sarcoplasmic reticulum, transverse tubule, and the sarcolemma of skeletal muscle [16]. In particular, inhibition of NOX activity in isolated skeletal myofibers significantly reduces exercise-induced cytosolic ROS production [17]. XO produces ROS as by-products of hypoxanthine oxidation. However, its relevance to human skeletal muscle is still debatable, as XO is not located in skeletal myofibers, but in endothelial cells that make up the blood vessels within skeletal muscle [18]. PLA2 contributes to elevation of ROS in skeletal muscle by (i) catalyzing production of arachidonic acid by ROS-producing lipoxygenases [19], (ii) promoting the translocation of NOX to the sarcolemma [20], and (iii) increasing ROS production in mitochondria [21]. Both calcium-dependent and calcium-independent isoforms of PLA2 participate in ROS production in skeletal muscle, but it is established that calcium-dependent PLA2 isoform is the major determinant of ROS production during exercise [21, 22]. Skeletal muscle also generates RNS, and, like ROS, its levels are elevated during muscle contraction [23]. Neuronal isoform of NOS (nNOS) is regarded as the primary source of RNS production in skeletal muscle and is located in cytoplasm and at the sarcolemma of skeletal muscle by forming part of the dystrophin-glycoprotein complex (DGC) [24].

## 3. Evidence for Oxidative Stress in Muscular Dystrophies

Muscular dystrophy (MD) is a collective term that refers to a group of genetically predisposed diseases that result in skeletal muscle weakness and degeneration [25]. Duchenne Muscular Dystrophy (DMD) is the most prevalent MD that affects an estimated 1 : 3500 males worldwide [26]. In the USA, an estimated 1 : 6000 boys are affected according to the Centers for Disease Control and Prevention (CDC) [27]. DMD is characterized by progressive skeletal muscle degeneration leading to early death due to respiratory or cardiac failure. The primary genetic cause of DMD is mutations in the* Dystrophin* gene resulting in dystrophin deficiency [28]. This in turn disrupts the DGC, resulting in structural destabilization and deregulated signaling, which subsequently lead to apoptotic and necrotic death of muscle cells [29]. Given the progressive nature of the disease, much effort has been put into identifying contributing factors, such as oxidative stress and increased calcium influx that are elevated in dystrophic muscles [11, 30–33]. The role of oxidative stress in pathology was implicated early by the observation that muscles from DMD patients contain a higher level of thiobarbituric acid-reactive products, which is indicative of lipid peroxidation brought about by oxidative stress. Dystrophic muscles also exhibit enhanced catalase, SOD, and glutathione reductase activity, which are reflective of oxidative stress [34]. In addition, 8-hydroxy-2′-deoxyguanosine (8-OHdG), a marker of free radical damage to DNA, was found to be elevated [33].

The* mdx* mouse that harbors a point mutation in the* Dystrophin* gene [35] has been widely studied as a mouse model for DMD. The muscles of* mdx* mice are histologically normal prior to the onset of necrosis at about 3 weeks of age, after which necrosis, inflammation, and subsequent regeneration ensue. However, unlike DMD patients, muscle damage subsides to a chronically low level by 8 weeks of age in* mdx* mice, with little impairment to muscle function, and lifespan is moderately reduced [30]. Interestingly, muscles from* mdx* mice exhibit increased levels of antioxidant enzymes prior to necrosis, indicative of a cellular response to oxidative stress [36]. Moreover,* mdx* muscles are more susceptible to oxidative stress-induced injury [37]. In agreement, ectopic expression of catalase in mitochondria was shown to increase lifespan and led to a partial restoration of* mdx* muscle function [38].

On the other hand, the exact contribution of ^∙^NO in DMD pathogenesis is unclear. The association of nNOS with the DGC and the consequential loss of membrane localization of nNOS in the* mdx* mice [39] raise the question of whether NOS-mediated oxidative stress is involved as well. Nevertheless, while several studies implicate correlation between NOS levels and the severity of DMD in different strains of* mdx* mice [32], prenecrotic* mdx* muscle fibers exhibit no change in ^∙^NO-induced nitrotyrosine formation. Moreover, NOS-null mice do not develop any dystrophic symptoms, and neither NOS-null nor* mdx* mice with ectopic NOS expression show any alteration in oxidative stress susceptibility [32]. Still, increased levels of oxidative stress markers and antioxidant enzyme expression in the* mdx* mice support the idea that oxidative stress may be causative of muscle degeneration in DMD. Furthermore, other types of MDs also exhibit signs of oxidative stress, which are discussed below.

Facioscapulohumeral muscular dystrophy (FSHD) is associated with the deletion of the D4Z4 macrosatellite repeats on chromosome 4q35, which increases expression of double homeobox 4 (DUX4) [40]. Muscle cells from FSHD patients show increased susceptibility to oxidative stress, augmented lipofuscin inclusions, elevated expression of antioxidant enzymes, and dysfunctional mitochondria [41].

Mutations in* Dysferlin* give rise to limb-girdle muscular dystrophy type 2B (LGMD2B) and Miyoshi myopathy (MM), as well as distal myopathy with onset in the tibialis anterior muscles. Dysferlin is enriched in the T-tubule of muscle fibers and plays an important role in maintenance of sarcolemma integrity and calcium influx [42].* Dysferlin*-null mice exhibit early elevation of reversible thiol oxidation and oxidative stress markers, implicating potential thiol oxidation of myogenic proteins [31].

Mutations in collagen VI causes two distinct MDs [43]: Bethlem myopathy (BM) which has a milder phenotype and Ullrich congenital MD (UCMD), which exhibits a more severe and rapid pathogenesis, thereby leading to an early death. Recent studies on both collagen VI-deficient mice and patients reported the evidence of defective autophagy, MAO-dependent oxidative stress, and resultant mitochondrial dysfunction attributing to pathogenesis of BM and UCMD [44].

Laminopathy is a collective term for a spectrum of age-related human diseases arising from mutations in the* LMNA* gene that encodes the intermediate filament nuclear lamin. It includes X-linked Emery-Dreifuss muscular dystrophy (EDMD) and sclerosing bone dysplasia [45]. The C-terminal cysteine tail of lamin A functions as a ROS sensor, the loss of which results in oxidative stress-driven premature cellular senescence [46]. Merosin (laminin-2) is located in the basal membrane of muscle fiber, and mutation of laminin *α*2 chain causes merosin-deficient congenital muscular dystrophy (MDCMD). It is characterized by neonatal muscle degeneration, colocalization of both necrotic and apoptotic signals in the patient muscle fibers, and increased autophagy.

## 4. Signaling Mechanisms Exploited by ROS

Many mechanisms have been proposed to explain muscle degeneration seen in MD. Among these, one of the most championed mechanism entails the loss of calcium homeostasis as a result of calcium influx through ion channels in the membrane [47]. The increase in cytoplasmic calcium is known to then regulate ROS primarily through disrupting mitochondrial function [21, 48], and the contribution of calcium to the muscular dystrophy pathology has been well-reviewed recently by Burr and Molkentin [49]. Below, we discuss alternative pathways that modulate and are modulated by ROS and how they are involved in muscular dystrophy.

### 4.1. NF-*κ*B Pathway

NF-*κ*B is a transcription factor that drives inflammatory gene expression [50]. NF-*κ*B activation occurs prior to the onset of muscular dystrophy in the muscles of* mdx* mice. Treatment with the antioxidant N-acetylcysteine (NAC) inhibits its activation, suggesting that oxidative stress lies upstream of NF-*κ*B and drives upregulation of NF-*κ*B to contribute to the myopathy in DMD [51, 52]. Consistent with this notion, treatment of* mdx* mice with IRFI-042, a synthetic vitamin E analogue, reduced NF-*κ*B DNA binding, TNF*α* expression, muscle necrosis, and enhanced regeneration [53]. Stretch-induced muscle damage in* mdx* muscles can be reduced by NAC treatment via reducing ROS and nuclear NF-*κ*B translocation [54]. In* mdx*;p65^+/−^ mice, muscle regeneration is improved, and this is correlated with hepatocyte growth factor (HGF) upregulation. Moreover, inhibition of HGF expression reversed the phenotype of* mdx*;p65^+/−^ mice, suggesting that the NF-*κ*B-HGF axis contributes to DMD pathogenesis [55]. Elevated TNF*α*, which activates NF-*κ*B, was also found in double mutants lacking both dystrophin and Stra13 [56].* mdx*/Stra13^−/−^ muscles were found to undergo oxidative stress-mediated degeneration. The degeneration of muscles was rescued by treatment of mice with NAC, further indicating a causal role for oxidative stress in muscle cell death. Altogether, it appears that oxidative stress-induced muscle degeneration is mediated, at least in part, by NF-*κ*B.

### 4.2. Autophagy in Muscle Degeneration

Autophagy is an evolutionarily conserved process in which the intracellular proteins and organelles are first engulfed into double membrane vesicles, termed as autophagosomes, and then delivered to lysosome for degradation [57]. Autophagy has been shown to be involved in various biological functions such as starvation adaptation, turnover of unfolded proteins and damaged organelles, cell metabolism, development, immunity, and cell death. Expectedly, emerging studies showed that autophagy also plays roles in the pathology of different human diseases including degenerative diseases, aging, and cancer [58]. Mechanistic or mammalian target of rapamycin complex 1 (mTORC1) and AMP-activated protein kinase (AMPK) are important autophagic regulators: mTORC1 inhibits autophagy via preventing the activation of ULK1, which is essential for the initiation stage of autophagy, while AMPK, a key sensor of cellular energy status to maintain energy homeostasis, has been shown to promote autophagy [59].

Autophagy plays a dual role in muscle homeostasis [60]. On one hand, excessive autophagy induced by overexpression of FOXO3 in myotubes causes atrophy via enhancing lysosomal proteolysis [61]. On the other hand, autophagy has been shown to be essential for the maintenance of muscle mass, as autophagy deficiency in muscle leads to abnormal mitochondria and muscle atrophy [62]. The role of autophagy in muscular dystrophies is complex. Defective autophagy is observed in DMD, BM, UCMD, and EDMD. In contrast, increased autophagy is detected in MDCMD [63]. Correspondingly, (i) in the* mdx* mouse, promotion of autophagy either by AMPK activation or by low protein diet ameliorates muscular dystrophy, which may be due to the elimination of defective mitochondria by mitophagy [64, 65]; (ii) in collagen VI-null mice, forced activation of autophagy by genetic, pharmacological, or dietary approaches is able to rescue the dystrophic phenotype [66, 67]; (iii) while in the laminin *α*2 chain-null dy3K/dy3K mouse model, inhibition of autophagy improves muscle morphology [68]. This suggests that the contribution of autophagy to pathology in different dystrophies is indeed distinct and that the forces driving muscle degeneration may be fundamentally different. Regardless, in dystrophies whereby impaired autophagy and accumulation of abnormal mitochondria have been found, the underlying mechanisms of how autophagy impairment occurs have not been well investigated. Studies by Pal et al. showed that the downregulation of autophagy in* mdx* skeletal muscle is caused by activated Src kinase, which is the key regulator of Nox2-mediated oxidative stress [69]. Both pharmacological and genetic inhibition of Nox2 or Src kinase induce autophagy, reduce oxidative stress, and improve pathophysiological abnormalities in* mdx* mouse muscles [69, 70]. However, one recent study showed that activation of P2RX7, an ATP-gated ion channel, increases autophagic flux in dystrophic myoblasts and myotubes, which contributes to nonapoptotic cell death [71]. This study is consistent with an earlier finding that inhibition of autophagy via activation of PI3K/Akt/mTOR pathway ameliorates dystrophic pathology in* mdx* mice [72].

Interestingly, epigenetic modifications including DNA methylation, histone modifications, and microRNAs have been shown to regulate autophagy [73]. For example, NAD^+^-dependent class III histone deacetylase Sirtuin 1 (Sirt1) positively regulates autophagy via interaction with autophagy genes (Atg5, Atg7, and Atg8) and direct deacetylation of these components [74]. A recent study showed that muscle-specific inactivation of the Sirt1 deacetylase domain leads to muscle developmental and regenerative defects [75]. Therefore, Sirt1 may contribute to muscle development through regulating autophagy. Despite these advances, much remains to be understood about the exact function of autophagy in muscle degeneration. In addition, a better understanding of its epigenetic regulation in dystrophies may provide targets for therapeutic intervention.

### 4.3. Telomere Shortening and Depletion of Satellite Cells

The observation that culturing human fibroblast in low oxygen conditions can extend the* in vitro* lifespan of the cells by slowing the rate of telomere shortening led to the idea that oxidative stress may contribute to telomere attrition. The telomeric repeat sequence is especially sensitive to oxidative damage, and single-stranded breaks induced by oxidative stress have been associated with telomere erosion [76]. In agreement, accelerated telomere loss is present in conditions of mitochondrial dysfunction where mitochondrial ROS are high [77].

Unexpectedly, while it has been shown that telomeres in muscle also shorten as a result of oxidative stress [78], there is no age-dependent loss of telomeres in muscles [79], possibly because the turnover of skeletal muscle is relatively low and satellite cells may not be used for homeostasis in normal uninjured muscle. In contrast, satellite cells in patients of muscular dystrophy are constantly activated for proliferation as a result of the distinctive degeneration-regeneration cycles. Early studies showed that myoblasts isolated from older DMD patients displayed lower proliferative capacity than those isolated from younger patients [80]. This drop in proliferative capability occurred due to replicative aging as a consequence of telomere shortening [81]. In recent times, landmark studies further support the role of telomere attrition in exhaustion of the satellite cell pool in muscular dystrophy.* mdx* mice lacking telomerase through the removal of the telomerase RNA component mTR, known as the* mdx*/mTR mice, recapitulate the severity of the dystrophic phenotype seen in humans that is not normally seen in the* mdx* mouse. The progressive worsening of pathology with age correlates with loss of proliferative capacity caused by telomere erosion in the myoblasts isolated from these mice [82, 83]. Similarly, reduced telomerase activity is responsible for the decline of the satellite cell pool that leads to the loss of muscle regeneration in the* mdx*/utrophin^−/−^ double knock-out mouse [84].

### 4.4. BMI-1: The Epigenetic Link to Oxidative Stress

Bmi-1 is a Polycomb group protein that prevents premature senescence by repressing the* INK4A/ARF* locus [85]. Of relevance, Bmi-1 controls the activity of metallothionein MT1, an antioxidant protein, in muscle to regulate oxidative stress, and increased Bmi-1 expression in muscle reduces the oxidative modification S-nitrosylation of MEF2C, which is known to inactivate MEF2C [86]. Consequently, loss of MEF2C function is causative of muscle degeneration [87].

## 5. Therapeutic Avenues

### 5.1. Physiological Role of ROS in Muscle Adaptation to Exercise

The observation that there was increased ROS production in the muscle of exercised rats by Davies et al. in 1982 [88] had led to the prediction that ROS might have a physiological role in exercise. Much research has gone into understanding this phenomenon, and now we know that ROS play multiple beneficial roles in exercise physiology. ROS can activate AMPK by reducing ATP, resulting in an increase in PGC-1*α* expression and activity after acute and long-term exercise [89]. PGC-1*α* is a master regulator of metabolic reprogramming that is key in muscle adaptation to exercise [90]. Thus treatment with NAC results in reduced AMPK and PGC-1*α* activation leading to lower uptake of glucose in an* in vitro* contraction model for exercise [91, 92]. Moreover, the elevation of myokine production upon exercise is blunted by treatment with antioxidants [93, 94]. A clinical trial performed by Ristow et al. [95] demonstrated that the beneficial effects of physical exercise mediated by a transient increase in ROS production leading to enhanced insulin sensitivity were abrogated by supplementation of the antioxidants vitamin C and vitamin E. These studies suggest that in healthy individuals, acute ROS production is required for adaption of skeletal muscle to exercise. However, chronic overproduction of ROS promotes oxidative stress that in turn contributes to a variety of muscular pathologies [96].

### 5.2. Antioxidant Therapies under Clinical Trials

Despite a prominent amount of evidence indicating a causal role of oxidative stress in the development of MDs, early clinical trials using antioxidants such as nicotinamide (vitamin B), tocopherols (vitamin E), and penicillamine did not bring about statistically significant clinical benefits [11]. Pentoxifylline (PTX) is a phosphodiesterase inhibitor with potent anti-inflammatory and antioxidant activity. Although a preclinical study on* mdx* mice showed significant muscle strength restoration [97], a recent clinical trial using PTX on DMD patients failed to yield any significant improvement on muscle strength and function [98].

One of the caveats for clinical trials using antioxidant therapy in muscle degenerative disorders such as DMD is that increased oxidative stress has to be targeted at very early stage of disease. Evidence of oxidative stress is detected in prenecrotic* mdx* mice [36]. As evidence accumulates to support that oxidative stress may precede necrotic cell death of muscle, clinical trials using antioxidant therapy should be initiated early to combat oxidative stress. A further point to note is that many antioxidants are nonspecific scavengers, such that they do not target a specific subcellular organelle source of ROS but are only able to remove existing ROS/RNS end products at the cellular level [99]. This is complicated by the physiological role of ROS as inducers of adaptive responses, which suggest that nonspecific targeting of ROS may unnecessarily suppress these cellular responses. This heightens the importance of searching for alternative routes of antioxidant therapy [100].

### 5.3. Targeting NF-*κ*B

Several pharmacological and natural products with antioxidant properties have been tested to combat the effects of elevated oxidative stress through inhibition of NF-*κ*B activity. Treatment of* mdx* mice with IRFI-042 diminished oxidative stress markers, reduced NF-*κ*B-dependent TNF-*α* expression, and relieved muscle fatigue [53]. Injection of* mdx* mice with curcumin, a pharmacological inhibitor of NF-*κ*B, improved histology and biochemical DMD features. Notably, inducible NOS (iNOS) levels were reduced in* mdx* mice, implicating a possible decrease in ^∙^NO-mediated oxidative stress [101]. Deferoxamine (DFX) is a potent iron-chelating agent that blocks iron-catalyzed ROS production and subsequent oxidative stress, with evidently reduced NF-*κ*B levels and improved muscle function in* mdx* mice [102]. Intense physical exercise aggravates muscle necrosis and is correlated with ROS in* mdx* mice [103]. The antioxidant NAC is effective for curbing NF-*κ*B expression in* mdx* mice, as well as ameliorating increased ROS production, protein thiol oxidation, loss of muscle contraction force, and serum creatine kinase (CK) level in* mdx* mice under prolonged exercise [31, 54]. A similar effect was observed when exercised* mdx* mice showed improved muscle function upon injection with nifedipine, a calcium channel blocker, with a concomitant reduction in mRNA expression of iNOS and NADPH oxidative subunits [104]. Angiotensin-converting enzyme (ACE) inhibitors curb proinflammatory and prooxidant activity of Angiotensin II. Treatment of* mdx* mice with the ACE inhibitor enalapril conferred a significant resistance to exercise-induced muscle weakening and a reduction in ROS production, improved limb muscle function, and reduced NF-*κ*B activation [105]. (−)-Epigallocatechingallate (EGCG), a polyphenol antioxidant compound from green tea extract, is known to reduce NF-*κ*B activity. Treatment of prenecrotic* mdx* mice with EGCG diminished expression of the oxidative stress marker lipofuscin and showed delayed necrosis of the extensor digitorum longus muscle [106, 107]. Melatonin is an endogenous anti-oxidant that scavenges ROS and RNS to reduce cellular redox status, and melatonin treatment on* mdx* mice was shown to improve muscle function [108]. In accordance with the preclinical data, 3-month administration of melatonin to DMD patients significantly reduced serum CK level, lipid peroxidation, nitrites, NF-*κ*B-driven inflammatory cascade [109], and curbed hyperoxidative status of erythrocytes in the treated patients [110]. Idebenone is a synthetic derivative of Coenzyme Q_10_ (CoQ), which is an electron carrier in the mitochondrial ETC; thus it acts as a potent antioxidant and inhibitor of lipid peroxidation by sequestering leaked electrons from the ETC [111, 112]. Initial studies using idebenone on* mdx* mice showed improved voluntary motion and cardiac function, thereby increasing survival [113]. A recent phase III clinical trial with idebenone improved respiratory function of 31 DMD patients [112], which counteracts the lethal failure of diaphragm muscle. However, this drug is useful only in patients who have not been previously treated with steroids [114].

### 5.4. Autophagy as a Therapeutic Avenue

Induction of autophagy via inhibition of mTORC1 by rapamycin has been reported to ameliorate dystrophic phenotype in 6-week-old* mdx* mice [115]. However, because of mTORC1 involvement in muscle regeneration, the potential usage of rapamycin as muscle therapeutics is limited [116]. Nevertheless, rapamycin nanoparticles, which are able to accumulate in the site of diseases, have been used to activate autophagy in* mdx* mice, and the results showed an improvement in physical performance of both skeletal and cardiac muscle [117]. AICAR (5-aminoimidazole-4-carboxamide-1-D-ribofuranoside), an established pharmacological activator of AMPK, has been used for the treatment of* mdx* mice. AICAR induces autophagy, enhances diaphragm mitochondria to resist calcium-induced permeability transition pore opening, and improves histopathology as well as muscle strength [64].

### 5.5. SIRT1 Stimulation

Resveratrol, a natural polyphenol from grapes and red wine, is known to induce SIRT1 expression [118]. Resveratrol treatment in* mdx* mice reduces nitrotyrosine, expression of NADPH oxidase subunits, and infiltration of fibrotic tissue in a SIRT1-dependent manner [119], although it may also act through inhibition of the acute inflammatory response to reduce the degenerative process [120].

### 5.6. Alternative Therapeutic Approaches

An alternative to pharmacological treatment could be direct supplementation of antioxidant proteins. As mentioned above, direct supplementation of melatonin has yielded a significant clinical outcome in DMD patients [109]. Creatine is a downstream product of glycine and arginine and exerts an antioxidant property by quenching aqueous ROS [121]. Creatine treatment inhibited muscle necrosis and enhanced mitochondria respiration capacity in* mdx* mice [122]. Four months of creatine administration to 30 DMD patients significantly improved their muscle function [123], although it was implicated that the therapeutic effect of creatine may be less effective for older patients [124]. Adenoviral overexpression of catalase in neonatal* mdx* mice was shown to be effective in reducing muscle impairment at an early phase of the disease [38]. This proposes the feasibility of an alternative genetic approach to combat elevated oxidative stress in DMD and potentially other muscle degenerative disorders. Nevertheless, one of the most significant problems plaguing the use of adenoviral-mediated therapy is the oxidation of the transgene mRNA due to elevated ROS production in dystrophic muscle, which hampers long-term efficacy of existing transgene therapies [125]. This highlights the importance of specialized protocols of transduction to achieve persistent expression in DMD patients. Satellite cell transplantation could be another strategy. Replacement of dystrophic myosatellite cells with Bmi-1 overexpressing satellite cells could be a way to restrain oxidative stress-induced muscle degeneration through MT1-mediated cellular proliferation and function [86]. Nevertheless, these alternative approaches of using cell therapy have their own pitfalls and still require technical refinement on cell delivery, minimization of cell death upon transplantation, and cytotoxicity of neighbouring cells in the niche [126].

## 6. Conclusion

**Figure 2:**
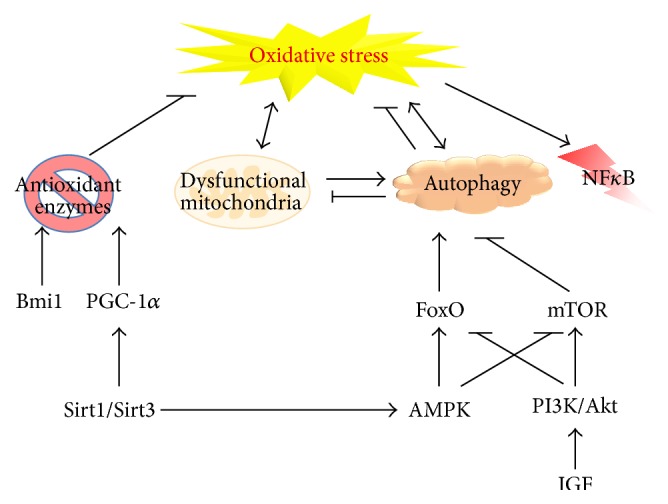
Mechanisms leading to oxidative stress in skeletal muscle. This schematic illustrates cellular mechanisms that are known to exert either prooxidant or antioxidant effects. Aberrant regulation of these pro- and antioxidative processes is indicated to play a role in muscle degenerative disorders. NF*κ*B: nuclear factor kappa-light-chain-enhancer of activated B cells; Bmi1: B lymphoma Mo-MLV insertion region 1 homolog; PGC-1*α*: peroxisome proliferator-activated receptor gamma coactivator 1-alpha; FoxO: forkhead box O; mTOR: mechanistic or mammalian target of rapamycin; AMPK: adenosine monophosphate-activated protein kinase; PI3K: phosphoinositide 3-kinase; IGF: insulin-like growth factor.

Studies from different animal models and patients indicate that elevated oxidative stress could be causative in inducing various signaling pathways which lead to muscle degeneration [11, 30, 31].  Figure 2 illustrates cellular mechanisms that cause excessive oxidative stress to trigger skeletal muscle degeneration in a variety of disorders. In degenerating muscle, increased oxidative stress is seen concurrently with elevated antioxidant enzyme level, probably as a measure to counteract excessive ROS. Important roles of epigenetic regulators such as Bmi-1 and Sirtuins in inducing antioxidant activity are also evident [86, 120]. Another cellular process that plays a pivotal role in redox homeostasis is autophagy. Aberrant autophagic activity disrupts muscle physiology either due to excessive lysosomal proteolysis or inefficient elimination of defective mitochondria, and correcting defective autophagy reduces oxidative stress [65]. Mitochondria are the major site of cellular ROS production, and various signaling pathways tightly regulate it. Hence deregulation of mitochondria plays a critical role in elevation of oxidative stress in degenerative muscle disorders [70]. One of the important downstream events of oxidative stress is activation of NF-*κ*B and its consequential inflammatory response, which is a key cellular event that contributes to muscle cell necrosis. These findings have been tested in preclinical and clinical trials to demonstrate their beneficial effects on delay of disease progression in dystrophic patients.  Table 1 summarizes pharmacological treatments through antioxidant therapies.

Nevertheless, whether oxidative stress is a causative factor or whether it is simply a by-product of muscle degeneration remains to be established unequivocally. The exact mechanisms through which distinct mutations in various degenerative disorders induce oxidative stress need to be elucidated. With an abundant list of drugs to target oxidative stress, a better understanding of the pathogenesis of the disorders will allow us to make informed decisions on the feasibility of using these drugs to treat dystrophies.

## Figures and Tables

**Table 1:** Pharmacological compounds used for antioxidant therapies in muscle degenerative disorders.

Compound	Predicted mode of action	Preclinical trial (mdx mouse)	Clinical trial
ACE inhibitors	Inhibits proinflammatory and prooxidant activity of Angiotensin II	Improved muscle function; enhanced resistance to exercise-induced muscle necrosis; reduced ROS production; reduced NF-*κ*B activity [105]	

AICAR	AMPK agonist; activates autophagy-mitophagy pathway	Improvement in diaphragm histopathology and force generation; normalized mitochondria calcium sensitivity [64]	

Creatine	Downstream product of glycine and arginine amino acids; quenches aqueous ROS	Improved muscle function; restored mitochondrial respiration capacity [122]	4-month administration to 30 DMD patients yielded significantly improved muscle function [123, 124]

Curcumin	Curcuminoid from turmeric; NF-*κ*B inhibitor	Improved sarcolemma integrity and muscle force; reduced TNF-*α* and iNOS levels [101]	

Deferoxamine	Iron-chelating agent	Reduced muscle damage and inflammatory response; reduced 4-hydroxynoneal and dihydroethidium staining [102]	

Epigallocatechin gallate	Polyphenol antioxidant compound from green tea extract	Improved muscle histology and physiology; reduced lipofuscin granules in diaphragm muscle; increased utrophin expression [106, 107]	

Idebenone	Short-chain analogue of Coenzyme Q; improves mitochondrial ETC function with antioxidant properties	Improved cardiac and running performance; reduction in inflammation and fibrosis [113]	52-week treatment on 31 DMD patients significantly improved respiratory function, albeit its efficacy is more prominent in patients who have not previously undergone steroid-treatment [112, 114]

IRFI-042	Synthetic vitamin E analogue; NF-*κ*B inhibitor	Partial restoration of limb strength and fatigue level; reduced oxidative stress; diminished NF-*κ*B-induced TNF-*α* expression [53]	

Melatonin	Endogenous ROS and RNS scavenger	Improved muscle function; reduced creatine kinase level; improved redox status of muscle [108]	3-month administration significantly reduced creatine kinase level, lipid peroxidation, nitrites, and NF-*κ*B-mediated inflammatory cascade; reduced hyperoxidative status of erythrocytes [109, 110]

N-acetylcysteine	Cysteine precursor; thiol-containing scavenger	Prevented exercise-induced muscle necrosis and elevation of creatine kinase level; reduced glutathione and protein thiol oxidation [31, 54]	

Nifedipine	Calcium channel blocker	Improved muscle function; enhanced resistance to exercise-induced muscle necrosis; reduction of iNOS and NADPH mRNA expression [104]	

Pentoxifylline	Phosphodiesterase inhibitor	Restored muscle strength; enhanced resistance to exercise-induced muscle necrosis; reduced creatine kinase level and ROS production [97]	12-month administration to 64 DMD patients produced no significant difference compared to the placebo group [98]

Rapamycin nanoparticles	mTORC1 inhibitor; activates autophagy	Increased skeletal muscle strength and cardiac contractile performance in both *mdx* and aged wild-type muscle [115–117]	

Resveratrol	Polyphenol in grapes and wine; SIRT1 activator	Improved muscle mass; reduced fibroblast infiltration; reduced nitrotyrosine [119]	
